# EPC-Derived Microvesicles Protect Cardiomyocytes from Ang II-Induced Hypertrophy and Apoptosis

**DOI:** 10.1371/journal.pone.0085396

**Published:** 2014-01-02

**Authors:** Shenhong Gu, Wei Zhang, Ji Chen, Ruilian Ma, Xiang Xiao, Xiaotang Ma, Zhen Yao, Yanfang Chen

**Affiliations:** 1 Department of Pharmacology and Toxicology, Boonshoft School of Medicine, Wright State University, Ohio, United States of America; 2 Department of Gerontology, the Affiliated Hospital of Hainan Medical College, Haikou, China; 3 Department of Cardiology, the People’s Hospital of Sanya, Sanya, China; 4 Department of Neurology, the Affiliated Hospital of Guangdong Medical College, Zhanjiang, Guangdong, China; Northwestern University, United States of America

## Abstract

Cell-released microvesicles (MVs) represent a novel way of cell-to-cell communication. Previous evidence indicates that endothelial progenitor cells (EPCs)-derived MVs can modulate endothelial cell survival and proliferation. In this study, we evaluated whether EPC-MVs protect cardiomyocytes (CMs) against angiotensin II (Ang II)-induced hypertrophy and apoptosis. The H9c2 CMs were exposed to Ang II in the presence or absence of EPC-MVs. Cell viability, apoptosis, surface area and β-myosin heavy chain (β-MHC) expression were analyzed. Meanwhile, reactive oxygen species (ROS), serine/threonine kinase (Akt), endothelial nitric oxide synthase (eNOS), and their phosphorylated proteins (p-Akt, p-eNOS) were measured. Phosphatidylinositol-3-kinase (PI3K) and NOS inhibitors were used for pathway verification. The role of MV-carried RNAs in mediating these effects was also explored. Results showed 1) EPC-MVs were able to protect CMs against Ang II-induced changes in cell viability, apoptosis, surface area, β-MHC expression and ROS over-production; 2) The effects were accompanied with the up-regulation of Akt/p-Akt and its downstream eNOS/p-eNOS, and were abolished by PI3K inhibition or partially blocked by NOS inhibition; 3) Depletion of RNAs from EPC-MVs partially or totally eliminated the effects of EPC-MVs. Our data indicate that EPC-MVs protect CMs from hypertrophy and apoptosis through activating the PI3K/Akt/eNOS pathway via the RNAs carried by EPC-MVs.

## Introduction

Pathological cardiac hypertrophy leads to heart failure which remains the major cause of cardiovascular morbidity and mortality [1]. Its pathology is characterized by cardiomyocyte (CM) hypertrophy, apoptosis and inflammation [2], [3]. It is well accepted that reactive oxygen species (ROS) plays an important role in the pathogenesis of cardiac hypertrophy [4]. Ang II-induced oxidative stress and inflammation have been demonstrated to contribute to the pathogenesis of cardiac hypertrophy [5], [6]. Some signaling cascades such as phosphatidylinositol-3-kinase (PI3K) and serine/threonine kinase (Akt) pathways may inhibit CM hypertrophy [7], [8]. The endothelial nitric oxide synthase (eNOS)/nitric oxide (NO) pathway, known as an important factor in regulating vascular function and one of the down-stream of Akt signaling, has also been shown to reduce ROS generation and exert anti-apoptotic effect on CMs [9], [10].

Cellular microvesicles (MVs) released from various cell types in response to different stimuli represent a novel way of cell-to-cell communication. Cellular MVs are functional because they transfer or deliver proteins and gene messages such as mRNA and microRNA (miRNA) to the target cells [11], [12]. Cellular MVs have been shown to reverse endothelial injury probably through their dual effects on NO and ROS production [13], [14]. It is suggested that bone marrow (BM)-derived endothelial progenitor cells (EPCs) could ameliorate cardiac hypertrophy [15], [16]. Of notes, emerging evidence suggest that EPC-MVs have cell protective features. They can increase Akt/eNOS protein expression and phosphorylation, and induce the expression of the anti-apoptotic protein Bcl-xL in target endothelial cells (ECs) [11]. EPC-MVs are also shown to reprogram hypoxic resident renal cells to regenerate [17] and to activate an angiogenic process in islet endothelium [18]. However, the effects of EPC-MVs on CM hypertrophy and apoptosis remains unclear.

In this study, we first determined the effects of EPC-MVs on Ang II-induced CM hypertrophy, viability and apoptosis. Then, we explored whether the underling mechanisms are associated with ROS production and PI3K/Akt/eNOS signaling pathway. In addition, we examined whether the effects of EPC-MVs were mediated by MV- carried RNAs.

## Materials and Methods

### Ethics Statement

Adult C57BL/6J genetic background mice were used in the present study to obtain BM-derived EPCs. The strains were maintained in our laboratory (22°C) with a 12-hr light/dark cycle and fed with standard chow and drinking water ad libitum. All experimental procedures were approved by the Wright State University Laboratory Animal Care and Use Committee and were in accordance with the Guide for the Care and Use of Laboratory Animals issued by the National Institutes of Health (NIH).

### Culture of Myocardial H9c2 Cell Line

H9c2 is a CM cell line (American Type Culture Collection, VA) derived from a clone of rat embryonic heart. Cells were cultured in Dulbecco’s Modified Eagle Medium (DMEM) supplemented with 10% fetal bovine serum (FBS) containing 100 U/ml of penicillin G and 100 µg/ml of streptomycin, in a humidified atmosphere containing 5% CO_2_ at 37°C. Before experimental intervention, confluent cultured cells were serum-starved for 12 h [9].

### Concentration-response Studies of Ang II on CMs

Ang II (Sigma-Aldrich, St. Louis, MO) induced H9c2 injury model was produced as previously reported [19]. In brief, H9c2 CMs were seeded in 12-well plates (5×10^4^ cells/well) or 96-well plate (5×10^3^ cells/well) during the logarithmic growth phase. When the cells were nearly 80% confluent, cells were incubated with different concentrations of Ang II (0, 10^−9^, 10^−8^, 10^−7^ and 10^−6^ M) for 24 h. After co-incubation, cells were collected for analyses (cell surface areas, viabilities and apoptosis). Upon the completion of this study, we chose 10^−6^ M of Ang II for the following studies.

### Culture of EPCs

The BM derived EPCs were cultured from adult (8–10 weeks of age, weight ranges from 25 g to 32 g) C57BL/6J genetic background mice as we previously described [20]. Mouse tibias and femurs were taken under deep anesthesia (pentobarbital, 150 mg/kg body weight) and BM was flushed out from tibias and femurs. BM mononuclear cells (MNCs) were isolated by using density gradient centrifuge method. After being washed with Phoshate-buffered saline (PBS), BM MNCs were counted and plated (1×10^7^ cells) on a 25 cm^2^ flask then grown in endothelial cell basal medium (EBM-2) supplemented with 5% FBS containing EPC growth cytokine cocktail (Lonza, Walkersville, MD). After 3 days in culture, non-adherent cells were removed by washing with PBS. Thereafter, culture medium was changed every 2 days.

### Preparation of EPC-MVs and RNA-free EPC-MVs

After being cultured for 7 d, EPC cultures were washed with PBS, and incubated with serum-free medium overnight. The conditional medium which contained EPC secretions was collected and centrifuged (1,000 *g*, 15 min) at 4°C. Then the supernatant was ultracentrifuged (100,000 *g*, 60 min) at 4°C to pellet EPC-MVs [20]. For preparation of RNA-free EPC-MVs, we disrupted the EPC-MVs with ribonuclease A (RNase A) [21], [22]. First, the EPC-MVs were incubated with 0.1% Triton X-100 (TX-100) for 5 min. Then the MV fraction was added in 200 U/ml of RNase A (Qiagen, CA) for 90 min at 37°C. After that, EPC-MVs were ultracentrifuged (100,000 *g*, 60 min) at 4°C to pellet the RNA deleted MVs (rdMVs) for the following experiments. To verify the effect of RNase A on MVs, the total RNAs were isolated from EPC-MVs and EPC-rdMVs using the RNA Isolation Kit (Ambion, NY), and the RNA concentrations were tested using quantitative assay (Thermo Scientific, Nanodrop 2000c, FL).

### Concentration-response Studies of EPC-MVs on CM Viability

To determine the effective EPC-MV dose for increasing CM viability, CMs were treated with Ang II (10^−6^ M) and different doses (0, 12.5, 25 and 50 µg/ml) of EPC-MVs. After 24 h, CMs were harvested for viability analysis. The protein concentration of EPC-MVs was quantified by using Bradford assay (Bio-Rad, Hercules, CA).

### Experimental Groups

Based on above studies, 10^−6^ M of Ang II and 50 µg/ml of EPC-MVs were used in the subsequent experiments. After reaching confluence, H9c2 CMs were randomly assigned to 4 different groups: serum-free medium (control), Ang II, Ang II+EPC-MVs, Ang II+drEPC-MVs. After incubation for 24 h, cells were harvested for analyses. For pathway blocking experiments, H9c2 CMs were pre-incubated with PI3K inhibitor (LY294002, 20 µM; Cayman Chemical, MI) or NOS inhibitor NG-nitro-arginine methyl ester (L-NAME, 100 µM; Sigma-Aldrich, St. Louis, MO) for 2 h [23].

### Detection of EPC-MV Merging with H9c2 CMs

For observing whether EPC-MVs could merge with H9c2 CMs, a lipid membrane-intercalating fluorescent dye (PKH26) was used to label EPC-MVs before co-incubation. Briefly, 50 µg/ml EPC-MVs was mixed with 2 ml of PKH26 (2×10^−6^ M; Sigma-Aldrich, St. Louis, MO) at room temperature (RT) for 5 min. The labeled mixtures were dialyzed in 2 ml of 1% bovine serum albumin (BSA) and ultracentrifuged at 100,000* g* for 60 min at 4°C to pellet the labeled MVs. After washed with EBM-2, the pellet was suspended with 1 ml of culture medium and added into H9c2 cells for 24 h incubation. The 4′, 6-diamidino-2 -phenylindole (DAPI, 1 ug/ml; Wako Pure Chemical Industries Ltd) was used for nuclear staining. Cell images were taken using an inverted microscope (EVOS, NY).

### Measurement of Cell Surface Area

The surface area of CMs in different groups was measured according to the method of Simpson [24]. In brief, cell images were captured by a 20×magnification digital inverted microscope. Then the images of CMs were traced and the cell surface areas were analyzed by using Image J software (NIH, MA). The surface areas of CMs in 6 different fields were averaged. The surface area data in each treatment group was presented as the rate of that in control group.

### Methyl Thiazolyl Tetrazolium (MTT) Assay

The viabilities of H9c2 CMs after different treatments were determined using the MTT Assay Kit (Invitrogen, NY) by following the manufacture’s protocol. The CMs culture was replaced with 100 µl of fresh culture medium. Cells in 96-well plate were added in 10 µl of 12 mM MTT solution and incubated at 37°C for 4 h. Then 100 µl of the sodium dodecyl sulfate (SDS)-HCl solution was added to each well and incubated at 37°C for 4 h. Finally, the 96-well plate was read by a microtiterplate reader (Packard) at 535 nm. The percentage of viability was defined as the relative absorbance of the treated cells versus the untreated controls [19].

### Flow Cytometry Analysis of Cell Apoptosis

The CM apoptosis was assessed by using an Apoptosis Assay Kit (Invitrogen, NY). The H9c2 CMs were collected by using 0.25% trypsin, and centrifuged at 200 *g* for 7 min. Cells were resuspended in 100 µl annexin-binding buffer, and incubated with 5 µl of annexin V-FITC and 1 µl of propidium iodide (PI) at RT in the dark for 15 min. Apoptotic cells were detected by a flow cytometer (Accuri C6 flow cytometer). The CMs stained with both annexin V and PI were considered to be late apoptotic CMs, and the cells stained only with annexin V were considered to be early apoptotic CMs [19].

### Immunohistochemistry of β-myosin Heavy Chain (β-MHC)

H9c2 CMs were fixed with 2% paraformaldehyde at RT for 30 min and then permeated with 0.1% TX-100 at RT for 15 min. After being blocked with 1% BSA and 2% donkey serum for 1 h, the cells were incubated with β-MHC antibody (1∶50; Millipore, MA) overnight at 4°C, and followed by incubation with Cy3-conjugated donkey anti-mouse antibody (1∶250; Jackson, PA) at RT in the dark for 1 h. DAPI was used for nuclear stain. Images were obtained with an inverted microscope.

### Intracellular ROS Detection

Intracellular ROS levels were determined by Dihydroethidium (DHE; Sigma-Aldrich, St. Louis, MO) staining [25], [26]. Cells were incubated with the DHE working solution (2 µM) at 37°C for 2 h. After that, the solution was replaced with fresh culture medium, and the cells were observed under an inverted microscope. The cells were then trypsinized and collected by centrifugation (200 *g*, 7 min). The percentage of DHE positive cells was measured by using flow cytometry method.

### Western Blot Analysis

After different treatments, proteins from H9c2 cells were obtained with lysis buffer (Thermo Scientific, FL) containing protease inhibitor. The proteins were subjected to electrophoresis and transferred onto nitrocellulose membranes. The membranes were blocked by incubating with 5% dry milk for 1 h, and then incubated with primary antibodies: against β-MHC (1∶1000; Sigma-Aldrich, St. Louis, MO), Akt (1∶500; Cell Signaling Technology, MA), p-Akt (Thr-308, 1∶500; Cell Signaling Technology, MA), eNOS (1∶500; Cell Signaling Technology, MA) or p-eNOS (ser-1177, 1∶500; Cell Signaling Technology, MA), at 4°C overnight. β-actin (1∶4000; Sigma-Aldrich, St. Louis, MO) was used to normalize protein loading. After being washed thoroughly, membranes were incubated with horseradish peroxidase (HRP) conjugated IgG (1∶40000; Jackson ImmunoResearch Labs, INC. PA) for 1 h at RT. Blots were then developed with enhanced chemiluminescence developing solutions and quantified [27].

### Statistical Analysis

Experimental data were expressed as the mean ± S.E, and were analyzed using one-way analysis of variance (ANOVA) followed by Bonferroni’s t-test. Values of *P*<0.05 were considered to be statistical significance.

## Results

### Effects of Ang II on H9c2 CM Cell Surface Area, Viability and Apoptosis

**Figure 1:**
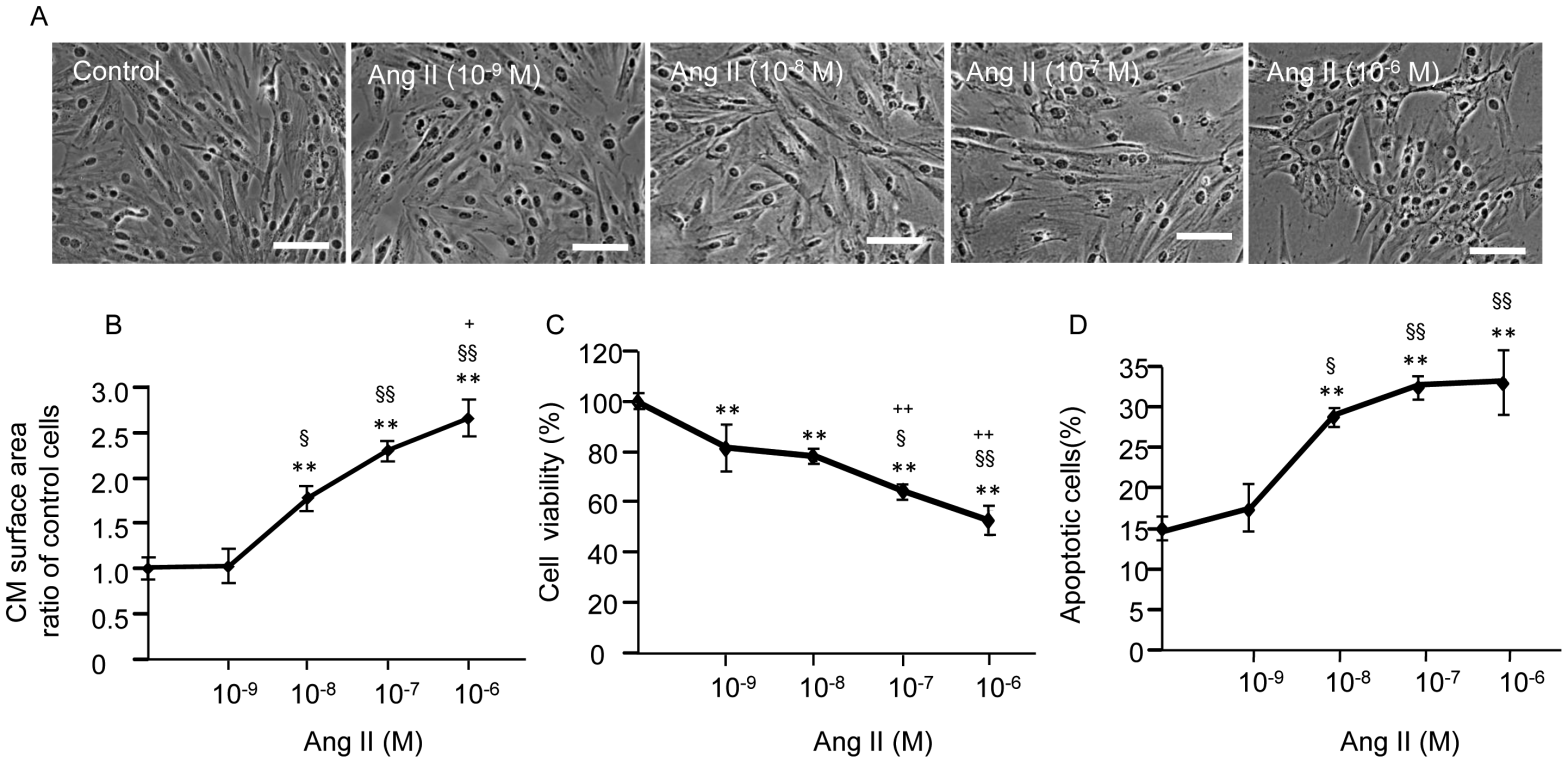
Concentration-response study of Ang II on H9c2 hypertrophy and apoptosis. (A) Representative images of H9c2 CMs. Scale bar, 100 µm. (B) Dose- dependent effect of Ang II on H9c2 cell surface area. (C) Dose-dependent effect of Ang II on H9c2 cell viability. (D) Dose-dependent effect of Ang II on H9c2 cell apoptosis. **P<0.01 *vs.* control; ^§^ *P*<0.05, ^§§^ *P*<0.01 *vs.* 10^−9^ M Ang II; ^+^ *P*<0.05, ^++^ *P*<0.01 *vs.* 10^−8^ M Ang II; n = 6/group.

To determine the effective dose of Ang II for inducing CM hypertrophy and apoptosis, H9c2 CM cells were treated with various concentrations (0, 10^−9^, 10^−8^, 10^−7^ or 10^−6^ M) of Ang II for 24 h. As seen in Figure 1A and 1B, Ang II dose-dependently increased cell surface area (*P*<0.05). The dose-dependent effects were also obtained in decreasing cell viability (*P*<0.01; Figure 1C) and in increasing cell apoptosis (*P*<0.05; Figure 1D). These results indicate the success of Ang II-induced CM hypertrophy and apoptosis model in H9c2 CMs. Based on these data, we chose 10^−6^ M Ang II for the following experiments.

### Effective Dose of EPC-MVs for Preventing Ang II-induced Reduction in CM Viability

**Figure 2:**
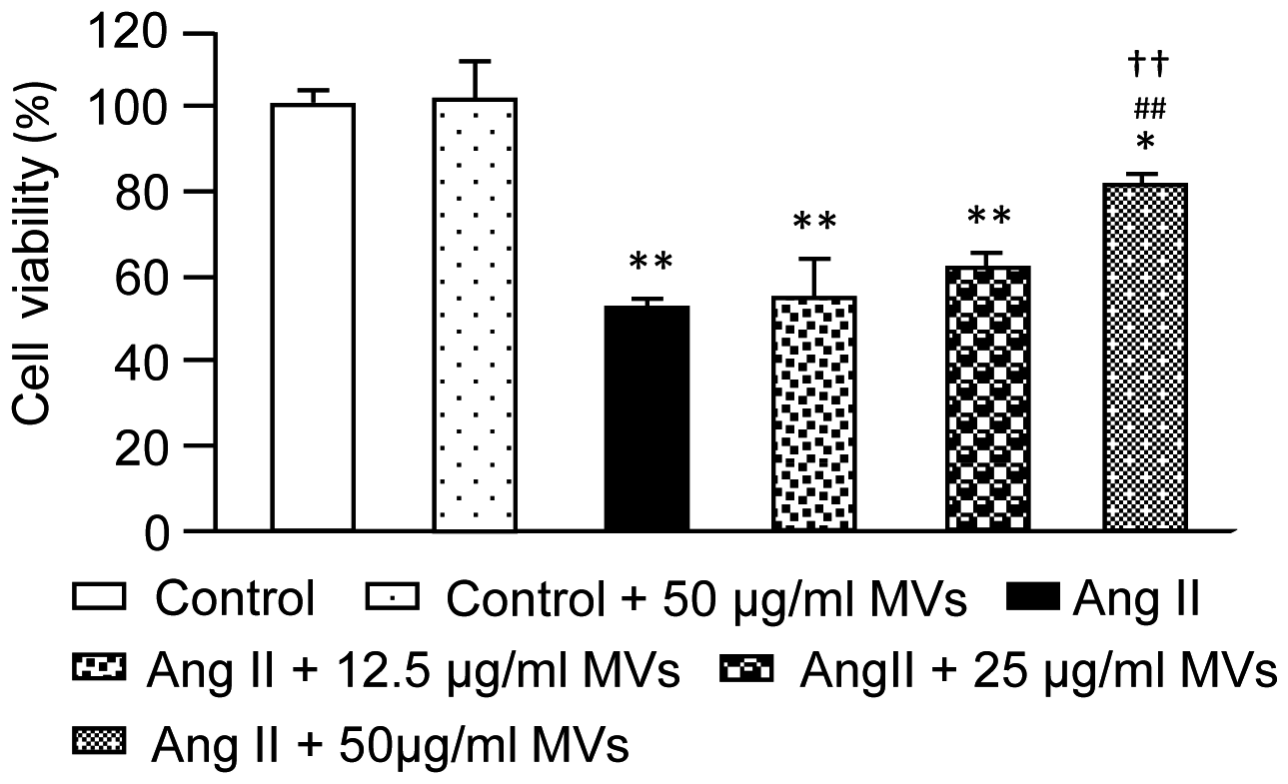
Dose-dependent effects of EPC-MVs on H9c2 viability. Summarized data on the effects of different EPC-MV doses on cell viabilities of H9c2 treated with 0 or 10^−6^ M Ang II. *P<0.05, **P<0.01 *vs.* control; ^##^ *P*<0.01 *vs.* Ang II, ^††^ *P*<0.01 *vs.* Ang II +25 µg/ml MVs; n = 6/group.

To determine the effective dose of EPC-MVs, we co-incubated different doses (0, 12.5, 25 or 50 µg/ml) of EPC-MVs and Ang II (10^−6^ M) with CMs for 24 h. We found that EPC-MVs at the dose of 50 µg/ml did not affect the survival of H9c2 cells, but significantly alleviated Ang II-induced reduction in CM viability (*P*<0.01; Figure 2). Thus, we chose 50 µg/ml EPC-MVs for the following experiments.

### EPC-MVs Merge with H9c2 CMs after Co-incubation

**Figure 3:**
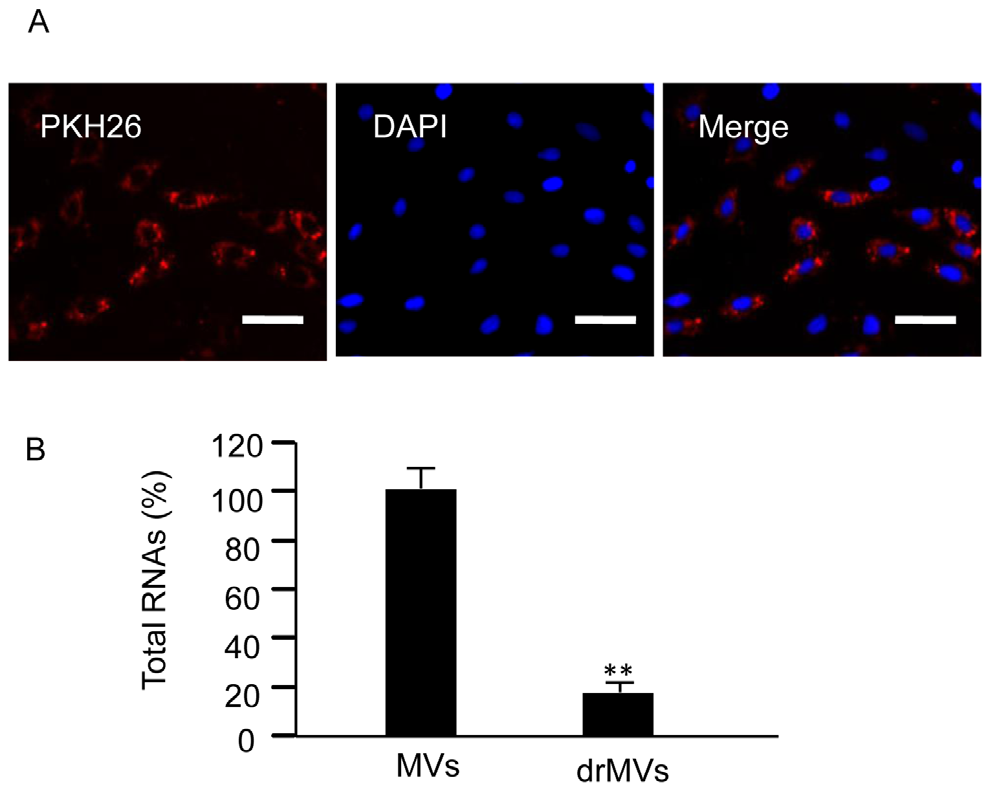
The incorporation of EPC-MVs with H9c2 and the RNAs depletion from EPC-MVs. (A) Representative images showing that EPC-MVs merge with H9c2 CMs. MVs were labeled with PKH26 (red). Nucleuses were labeled with DAPI (blue). Scale bar, 100 µm. (B) Summarized data of total RNAs in MVs and rdMVs. RNase treatment is effective in depleting RNAs from EPC-MVs. **P<0.01, EPC-rdMVs *vs.* EPC-MVs; n = 3/group. rdMVs: RNA deleted MVs.

The PKH26 labeled EPC-MVs were co-incubated with H9c2 CMs for 24 h. The PKH26 fluorescent was able to be detected in the cytoplasm of the H9c2 CMs, suggesting that EPC-MVs could merge with H9c2 CMs (Figure 3A).

### RNase Treatment Effectively Depletes RNAs from EPC-MVs

To investigate the possible role of EPC-MV carried RNAs in MV function, we digested the total RNAs inside MVs by using RNAse A. As expected, we found that RNase A was able to deplete more than 80% of total RNAs in EPC-MVs (100±10.1% and 17.5±3.5%, *P*<0.01, MVs *vs.* rdMVs; Figure 3B).

### EPC-MVs Decrease Ang II-induced CM Hypertrophy via their Carried RNAs

**Figure 4:**
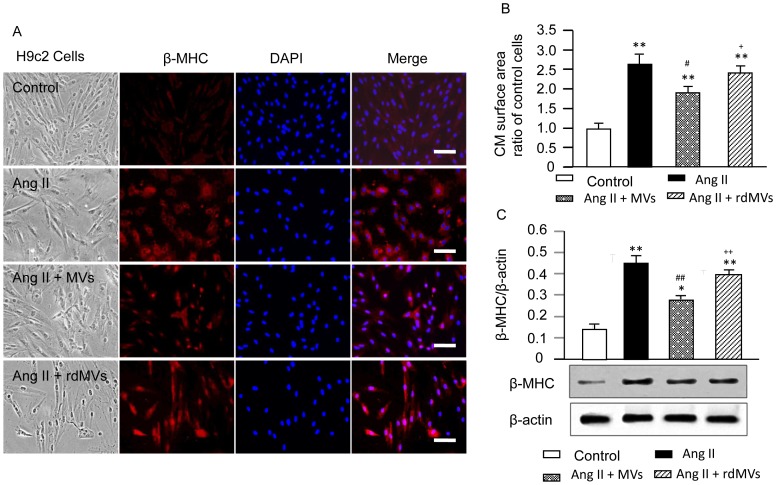
Effects of EPC-MVs on Ang II-induced CM hypertrophy and β-MHC protein expression. (A) Representative immunohistochemistry images of β-MHC expression in H9c2 CMs in each group. H9c2 CMs were labeled with β-MHC antibody (red), and DAPI (blue, for nucleus). Scale bar, 100 µm. (B) Summarized data of surface areas of CMs in each group. (C) Western blot bands and graphs showing the β-MHC expression in H9c2 CMs in different treatment groups. The molecular weights are 223 kDa for β-MHC and 43 kDa for β-actin. *P<0.05, **P<0.01 *vs.* control, ^#^ *P*<0.05, ^##^ *P*<0.01 *vs.* Ang II, ^+^ *P*<0.05, ^++^ *P*<0.01 *vs.* Ang II+ MVs; n = 4/group. rdMVs: RNA deleted MVs.

Ang II induced CM hypertrophy is characterized by cell size increase and activation of fetal cardiac genes such as β-MHC [28]. Here, we found that EPC-MVs decreased Ang II-induced CM enlargement in cell surface area (1.9±0.4 and 2.7±0.2, Ang II+MVs *vs.* Ang II, *P*<0.01; Figure 4A and 4B) and up-regulation in β-MHC expression (*P*<0.01; Figure 4A and 4C). In contrast, EPC-rdMVs totally blocked these effects (*P*<0.01; Figure 4).

### EPC-MVs Protect CMs from Ang II-induced Decrease in Viability Partially via their Carried RNAs which Activate PI3K/NOS Pathway

**Figure 5:**
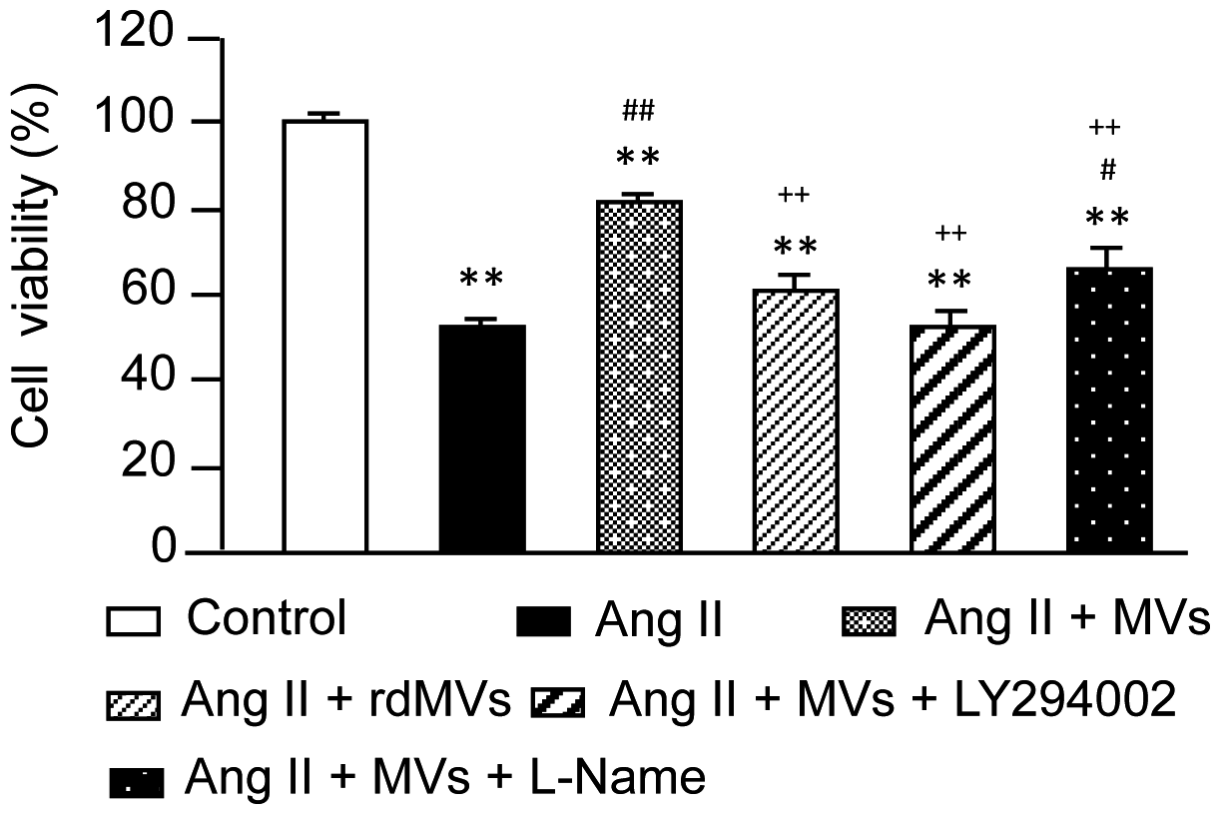
Effect of EPC-MVs on cell viability of Ang II-treated H9c2 CMs. Summarized data on H9c2 CM viability in each group. **P<0.01 *vs.* control; ^#^ *P*<0.05, ^##^ *P*<0.01 *vs.* Ang II; ^++^ *P*<0.01 *vs.* Ang II+EPC-MVs; n = 6/group. rdMVs: RNA deleted MVs.

Fig. 5 shows co-incubation of EPC-MVs prevented Ang II-induced decrease in cell viability (81.2±2.6% and 52.2±2.1%, Ang II+MVs *vs.* Ang II, *P*<0.01). EPC-rdMVs were less effective on improving H9c2 cell viability compromised by Ang II (61.3±3.3% and 81.2±2.6%, Ang II+EPC-rdMVs *vs.* Ang II+EPC-MVs, *P*<0.01), suggesting that the RNAs carried by EPC-MVs were partially required for the protective effect. In addition, LY294002 could abolish and L-NAME partially blocked the protective effect of EPC-MVs (52.8±3.7%, 65.7±4.6% and 81.2±2.6%, MVs+LY294002 or MVs+L-NAME *vs.* MVs, *P*<0.01). These demonstrate the involvement of PI3K/NOS pathway in the protective effect of EPC-MVs.

### EPC-MVs Protect CMs from Ang II-induced Apoptosis via their Carried RNAs which Activate the PI3K/NOS Pathway

**Figure 6:**
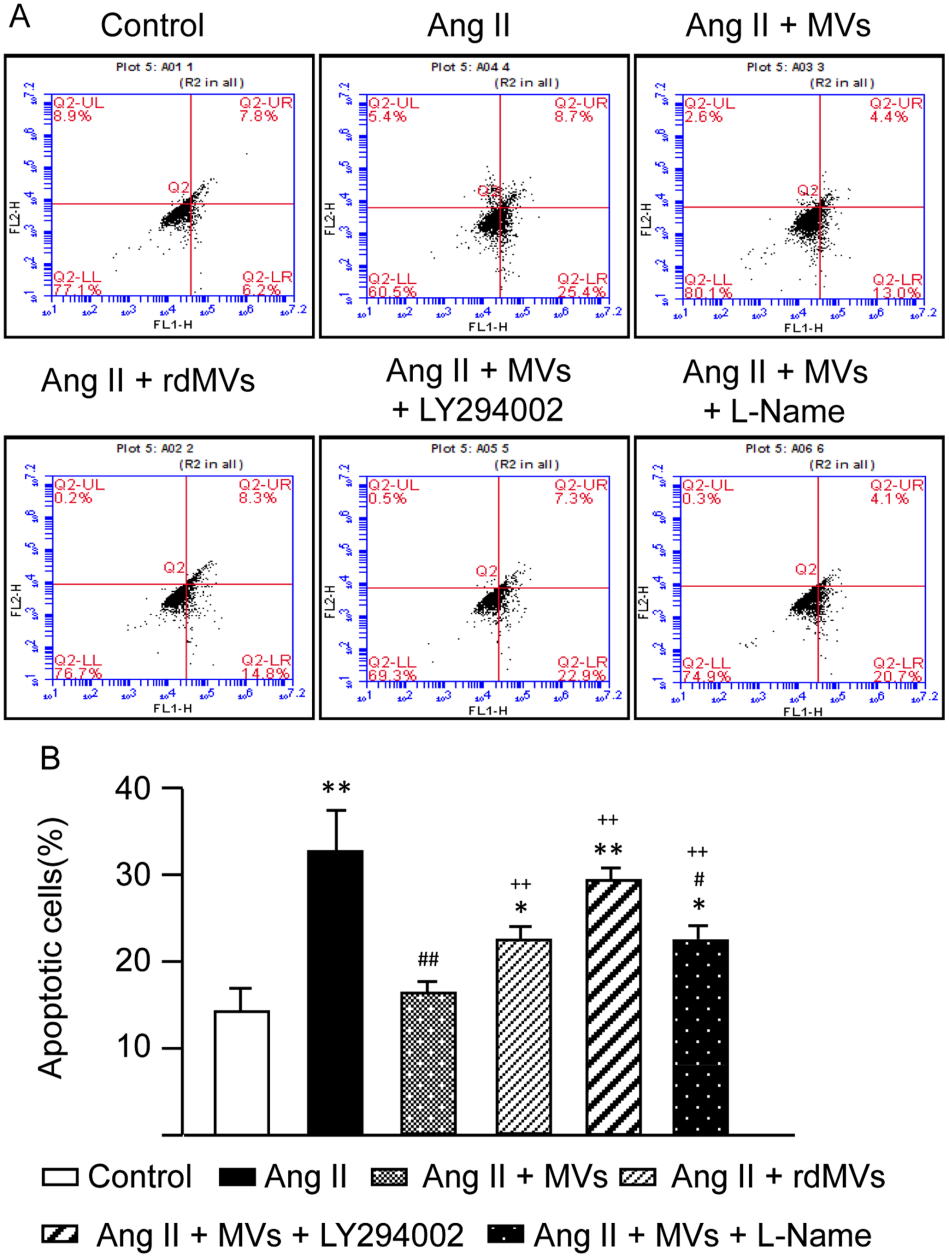
Effect of EPC-MVs on Ang II-induced CM apoptosis. (A) Representative flow cytometric plots of H9c2 CM apoptosis in different treatment groups. (B) Summarized data on the percentage of apoptotic H9c2 CMs in each group. **P<0.01 *vs.* control, ^#^ *P*<0.05, ^##^ *P*<0.01 *vs.* Ang II, ^++^ *P*<0.01 *vs.* Ang II+MVs; n = 6/group. rdMVs: RNA deleted MVs.

As shown in Figure 6, EPC-MVs significantly decreased Ang II-induced CM apoptosis (16.1±1.2% and 32.8±4.5%, Ang II+MVs *vs.* Ang II, *P*<0.01). This effect was significantly reduced in the EPC-rdMV group (22.1±1.6% and 16.1±1.2%, Ang II+rdMVs *vs.* Ang II+MVs, *P*<0.01). Furthermore, LY294002 could abolish and L-NAME partially blocked the protective effect of EPC-MVs (29.1±1.7%, 22.4±1.4% and 16.1±1.2%, MVs+LY294002 or MVs+L-NAME *vs.* MVs, *P*<0.01).

### EPC-MVs Inhibit Ang II-induced ROS Overproduction in CMs via their Carried RNAs which Activate PI3K/NOS Pathway

**Figure 7:**
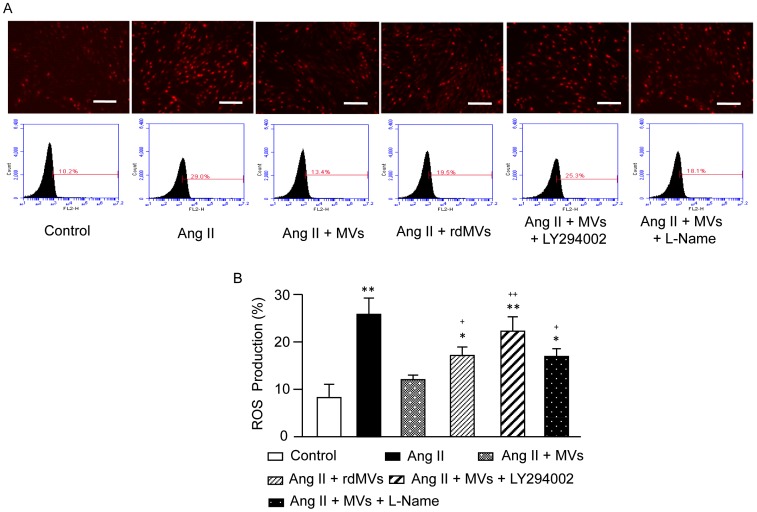
Effect of EPC-MVs on intracellular ROS production of Ang II-treated H9c2 CMs. (A) Representative images of intracellular DHE staining and flow traces in different groups. Scale bar, 200 µm. (B) Summarized data on the measurement of ROS production in H9c2 CMs in different groups. *P<0.05, ** *P*<0.01 *vs.* control, ^##^ *P*<0.01 *vs.* Ang II, ^+^ *P*<0.05, ^++^ *P*<0.01 *vs.* Ang II+MVs; n = 6/group. rdMVs: RNA deleted MVs.

Oxidative stress is one of the main contributing factors that initiate hypertrophy and apoptosis in Ang II treated CMs [28]. We examined the role of EPC-MVs on ROS overproduction in CMs induced by Ang II. As shown in Figure 7A, Ang II induced an increase in DHE positive cells, which was suppressed by EPC-MVs. The flow cytometric data also showed that EPC-MVs significantly suppressed Ang II-induced intracellular ROS overproduction (Figure 7B). EPC-rdMVs were less effective on reducing Ang II-induced ROS overproduction in CMs (*P*<0.05). In addition, pre-incubation with LY294002 or L-NAME could abolish or partially block the protective effect of EPC-MVs (*P*<0.01; Figure 7).

### EPC-MVs Activate PI3K/Akt/eNOS Signaling Pathway in CMs

**Figure 8:**
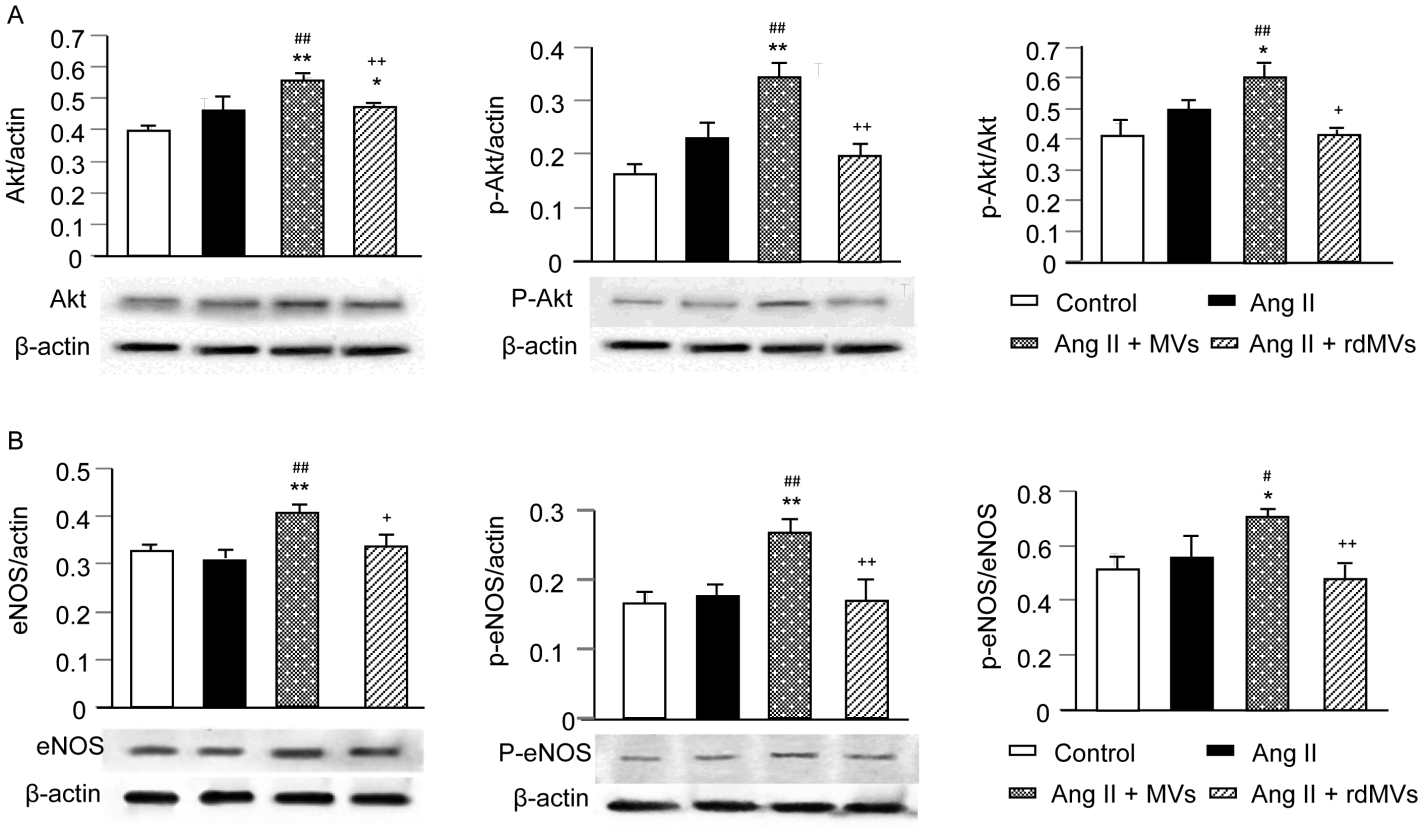
EPC-MVs up-regulate Akt/eNOS activation in Ang II-treated H9c2 CMs. Representative Western blot bands showing Akt/p-Akt (A), and eNOS/p-eNOS (B) expression of CMs in different treatment groups. The molecular weights are 60 kDa for Akt and p-Akt, and 140 kDa for eNOS and p-eNOS. Summarized data on Akt/p-Akt (A) and eNOS/p-eNOS (B) expression in CMs in different treatment groups. *P<0.05, **P<0.01 *vs.* control, ^#^ *P*<0.05, ^##^ *P*<0.01 *vs.* Ang II, ^+^ *P*<0.05, ^++^ *P*<0.01 *vs.* Ang II+MVs; n = 4/group. rdMVs: RNA deleted MVs.

Akt phosphorylation has been demonstrated to reflect Akt activation. EPC-MVs significantly increased the protein expression of p-Akt/Akt (*P*<0.01; Figure 8A) and p-eNOS/eNOS (*P*<0.01; Figure 8B) in CMs. EPC-rdMVs were less effective on up-regulating Akt, p-Akt, eNOS and p-eNOS (*P*<0.05 or 0.01; Figure 8).

## Discussion

In this study, we demonstrated for the first time that EPC-MVs protect CMs from Ang II-induced hypertrophy and apoptosis. The underlying mechanism may partially rely on the RNAs carried by EPC-MVs which could inhibit ROS overproduction and activate the PI3K/Akt/eNOS signaling pathway.

MVs were first described about 30 years ago and considered to be membrane nano-fragments (0.05–1 µm) [13]. MVs are shed from the cell surface upon activation, stress or apoptosis. It can be derived from various cell types, such as platelets, endothelial cells, EPCs and leukocytes, etc [29], [30]. They express different cell surface markers, which vary according to their cell origin, and the process of MV formation. Therefore, MVs can be used as biomarkers for disease and indicators for therapeutic efficacy. More recently, studies showed that MVs exert effects on anti-inflammatory, anticoagulant and angiogenesis [31]. Our previous study demonstrated that circulating MVs from db/db diabetic mice impair the EPC function *in* *vitro* and *in* *vivo* [20]. EPCs have been shown to have beneficial effects on cardiovascular regeneration and protection [32]–[34]. MVs released from EPCs could carry their parent cell biological information and thus are functional to the target cells [13]. For examples, EPC-MVs trigger a repair program to injured tissues such as vasculatures, kidney and pancreatic islets [11], [17], [18]. Therefore, targeting the functional properties of EPC-MVs could open a novel therapeutic approach for vascular disease. However, there is no information regarding the effects of EPC-MVs on cardiac hypertrophy and apoptosis.

For testing our hypothesis that EPC-MVs play a protective role in cardiac hypertrophy and apoptosis, we produced the model of Ang II-induced CM hypertrophy and apoptosis as previously reported [35], [36]. As we expected, Ang II dose-dependently induced CM hypertrophy and apoptosis, suggesting a success of model reproduce. After co-incubation of EPC-MVs with CM, we found that EPC-MVs can effectively incorporate into CMs. This finding is in agreement with previous reports showing that the MVs can merge with CMs or ECs [11], [37]. Most of the previous studies on EPC-MVs are focusing on angiogenesis. For examples, EPC-MVs are able to trigger *in* *vivo* angiogenesis in a murine model of hindlimb ischemia [11]. Incubation of EPC-MVs with HUVECs promotes EC survival, proliferation and *in* *vitro* formation of capillary-like structures [11]. Here, we demonstrate for the first time that EPC-MVs prevent CMs from Ang II-induced hypertrophy and apoptosis.

The underlying mechanisms of EPC-MVs’ protective effects on CMs might involve oxidative stress and PI3K/Akt/eNOS signaling pathway. Firstly, ROS overproduction has been demonstrated in Ang II-treated CMs by others [36], [38] and in our present study. Meanwhile, our results reveal that the anti-hypertrophic and anti-apoptotic effects of EPC-MVs are correlated with the inhibition of ROS overproduction. Secondly, we found in this study that EPC-MVs up-regulate Akt/eNOS and p-Akt/p-eNOS expression in CM hypertrophy model. The PI3K/Akt signaling pathway has been shown to play a crucial role in protecting CMs from Ang II-induced hypertrophy and apoptosis [39], [40]. In particular, it is indicated that EPC-MVs are involved in the angiogenic and anti-apoptotic program by shuttling specific RNAs associated with PI3K/Akt and eNOS pathways [11]. Supported by these previous studies, our data suggest that activation of PI3K/Akt/eNOS pathway could be responsible for the effects of EPC-MVs on preventing CMs from Ang II-induced hypertrophy and apoptosis. Thirdly, activation of eNOS/NO production scavenges superoxide anion to prevent ROS overproduction [41], [42]. These studies provide mechanism explanations for our novel findings that EPC-MVs activate eNOS and reduce ROS production in Ang II-treated H9c2 CMs. Finally and most importantly, we have applied the pathway inhibitors to verify the role of PI3K/Akt/eNOS pathway in EPC-MVs’ protective effects. We found that the pathway blockers partially (L-NAME) or totally (LY294002) inhibit the protective effects of EPC-MVs on Ang II-induced CM hypertrophy, apoptosis and oxidative stress. Taken together, our results demonstrate that EPC-MVs could trigger the PI3K/Akt/eNOS signaling cascades to reduce ROS production, and consequently to inhibit Ang II-induced hypertrophy and apoptosis.

MVs exert different functions depending on their composition, such as protein, receptor, mRNA and miRNA. A recent report suggests that RNAs in MVs have led to the genetic communication between cells, and the mRNAs in these RNAs could be translated into proteins after being taken up by the cells [37]. Deregibus et al showed that EPC-MVs activate angiogenic program in ECs by a horizontal transfer of mRNA [11]. In the present study, we investigated the role of EPC-MVs carried RNAs in the effects of EPC-MVs. Interestingly, our data showed that the protective effects of EPC-MVs on CM apoptosis, cell viability and ROS production could be partially blocked by RNA depletion. Depletion of RNAs abolished EPC-MVs’ effects on hypertrophy and modulating Akt/eNOS signaling pathway, suggesting that the other mechanisms such as their carried protein components might also be involved. Our findings suggest that the beneficial effects of EPC-MVs are partly mediated by their carried RNAs. Nevertheless more detailed mechanisms, such as the responsive miRNAs, mRNAs and/or proteins, await future exploration.
